# In Vitro Antiparasitic Activity of Propyl-Propane-Thiosulfinate (PTS) and Propyl-Propane-Thiosulfonate (PTSO) from *Allium cepa* against *Eimeria acervulina* Sporozoites

**DOI:** 10.3390/microorganisms10102040

**Published:** 2022-10-15

**Authors:** María Arántzazu Aguinaga-Casañas, Nuria Mut-Salud, Ana Falcón-Piñeiro, Ángela Alcaraz-Martínez, Enrique Guillamón, Alberto Baños

**Affiliations:** 1 Department of Microbiology and Biotechnology, DMC Research Center, Camino de Jayena s/n, 18620 Granada, Spain; 2 Department of Microbiology, University of Granada, Fuente Nueva s/n, 18071 Granada, Spain

**Keywords:** *Eimeria*, sporozoites, coccidian, *Allium*, PTSO, PTS, MDBK, onion

## Abstract

Among the alternatives to control avian coccidiosis, alliaceous extracts stand out due to their functional properties. Despite this, most of the references are focused just on garlic. In this study, we analyze the in vitro effects of propyl-propane thiosulfinate (PTS) and propyl-propane thiosulfonate (PTSO), two organosulfur compounds from onion, on MDBK cells infected with sporozoites of *Eimeria acervulina*. To this aim, two different experiments were performed. In the first experiment, sporozoites were previously incubated for 1 h at 1, 5 and 10 µg/mL of PTS or PTSO and added to MDBK cells. In the second experiment, MDBK cells were first incubated for 24 h at different concentrations of PTS or PTSO and then infected with *E. acervulina* sporozoites. Then, 24 h after inoculation, the presence of *E. acervulina* was quantified by qPCR. MDBK viability was measured at 72 h post-infection. Sporozoites incubated at 10 µg/mL of PTS and PTSO inhibited the capability to penetrate the cells up to 75.2% ± 6.44 and 71.7% ± 6.03, respectively. The incubation of MDBK with each compound resulted in a preventive effect against sporozoite invasion at 1 µg/mL of PTS and 1 and 10 µg/mL of PTSO. Cells incubated with PTSO obtained similar viability percentages to uninfected cells. These results suggest that the use of PTS and PTSO is a promising alternative to coccidiosis treatment, although further in vivo studies need to be performed.

## 1. Introduction

Avian coccidiosis constitutes one of the most important health problems that causes high economic losses to the poultry industry throughout the world [1,2]. Current treatments using drugs entail high costs and the appearance of resistant pathogens; therefore, alternative anti-coccidian strategies are needed [3]. In recent years, numerous alternatives have been proposed, including the use of vaccines and natural products that comprise fatty acids, antioxidants, fungal and, especially, herbal extracts [4,5,6]. Their use as feed additives presents several advantages due to their multiple benefits, such as the absence of a withdrawal period, appetite-stimulating action and immunomodulatory and antimicrobial properties [7].

Botanical extracts, often applied as feed additives in the form of essential oils (EOs) contain numerous secondary metabolites with functional roles, such as protecting plants from herbivores, insects, microbial infections and other challenges [8]. These natural bioactive compounds can be isolated from many different natural sources, including roots, herbs and bulbs, and have shown to exert positive effects on animal growth and health due to their antimicrobial, antiviral, antifungal and antioxidant properties [9,10].

Among the different phytogenic plant extracts, those derived from *Allium* spp. hold large promise due to their variety of bioactive compounds including polyphenols, saponins, fructans, organosulfur compounds among others, being one of the most studied plants of medicinal importance [11]. Several studies have reported that the addition of *Allium* spp. in different forms to poultry diets improves productivity, immune response, gut ecosystem and lipid metabolism of animals [12]. In addition, *Allium* compounds have shown antiparasitic activity against intracellular protozoa, including *Eimeria* spp. and *Leishmania* [13,14].

The most important class of bioactive compounds in *Alliaceae* is organosulfur compounds (OSCs), which are synthesized during tissue damage as part of the defense mechanism against external challenges. However, most of the literature focuses on garlic (*Allium sativum*) and its main bioactive compound, allicin, whose low stability hinders its use as a feed additive [15]. Moreover, many studies are based on garlic extract, a nonspecific term as their compounds differ depending on the fermentation processes of plants, extraction methods, etc. [16]. For this reason, it is necessary to reach a consensus on the use of a feed additive based on *Allium* that preserves its bioactive compounds. In this sense, propyl propane thiosulfonate (PTSO) and propyl propane thiosulfinate (PTS) from onion (*Allium cepa*) have shown a wide variety of functional properties, as well as greater stability and bioavailability compared to other OSCs, which makes them suitable for use in the feed sector [17]. PTS is formed from propiin (S-propyl-L-cysteine sulfoxide) due to the action of aliinase and through dismutation or disproportionation reactions is transformed into PTSO [18].

The effects of PTS or PTSO when added to the diet of farm animals have been previously studied in chickens and laying hens, disclosing their capability to improve productive parameters and modulate gut microbiota by reducing enteropathogens such as *Salmonella enterica* and *Escherichia coli* while respecting those beneficial bacteria groups such as *Bifidobacterium* spp. and *Lactobacillus* spp. [19,20,21]. These effects have also been observed in piglets and fattening pigs [22,23]. Moreover, PTS and PTSO showed antibacterial activity against Gram-negative and Gram-positive multidrug-resistant bacteria isolated from human samples, as well as antifungal activity against *Candida* spp. [24,25].

Despite the fact that the antibacterial activity of PTS and PTSO is very well described, there is little information about their antiparasitic properties. In this study, we evaluated the in vitro effects of these OSCs on the capability of *E. acervulina* sporozoites to infect MDBK cells and the preventive capacity of each compound against intracellular sporozoite invasion.

## 2. Materials and Methods

### 2.1. Allium Compounds and Reagents

PTS and PTSO (95% purity) were supplied by DOMCA SAU (Granada, Spain). Each compound was previously dissolved in dimethyl sulfoxide (DMSO) to a final concentration of 1 mg/mL, and from these stock solutions, the different conditions to be tested were prepared using Dulbecco′s Modified Eagle′s Medium (DMEM). All reagents were purchased from Sigma-Aldrich Quimica S.L. (Madrid, Spain), unless otherwise indicated.

### 2.2. Parasites

*E. acervulina* oocysts were isolated from field samples of unvaccinated chicken feces on a collaborating farm.. Briefly, fresh samples were subjected to centrifugation at 8000× *g* for 5 min using 50% sodium hypochlorite (NaClO) floatation solution [26]. Isolated oocysts were then sporulated using 2.5% potassium dichromate (K_2_Cr_2_O_7_). The presence of *E. acervulina* was confirmed by morphological analysis under optical microscopy by using the software tool COCCIMORPH (http://www.coccidia.icb.usp.br/coccimorph (accessed on 7 October 2021)), [27], after which they were stored at 4 °C. The oocysts were then washed with PBS from K_2_Cr_2_O_7_ in phosphate-buffered saline (PBS, pH = 7.2). Surface sterilization using 2% sodium hypochlorite for 15 min was then performed followed by a new washing with sterile PBS. For excystation, oocysts walls were broken using 0.5 mm sterile glass beads (Sigma-Aldrich, Madrid, Spain) in sterile distilled water at pH 3.0, and the released sporocysts were subjected to enzymatic excystation using a hatching solution at pH 8.0 consisting in 0.25% trypsin (w/v), 9.8% Hanks’ balanced salts (w/v) and 1% sodium taurocholic acid (w/v) (Alfa Aesar, Kandel, Germany), at 41 °C for 90 min [28]. Finally, sporozoites were purified by filtration using a 10 µm pluriStrainer^®^ filter (PluriSelect, Leipzig, Germany), washed with sterile PBS and counted using a hemocytometer.

### 2.3. Cell Cultures

Madin-–Darby bovine kidney (MDBK) cells (DSMZ, German Collection of Microorganisms and Cell Cultures, Braunschweig, Germany) were used as host cell cultures for *E. acervulina*. MDBK cells were maintained in completed medium (DMEM supplemented with 10% FBS and 1% penicillin-streptomycin) at 37 °C, 5% CO_2_ and humidity. For the different experiments, they were seeded in 24-well plates (Thermo Fisher Scientific, Roskilde, Denmark) at a density of 5 × 10^4^ and grown overnight to obtain approximately a confluency of 80–90% at the time of inoculation with *Eimeria* parasites.

### 2.4. Cytotoxicity Assays

To evaluate the effect of onion compounds on sporozoite viability, aliquots of 2.5 × 10^3^ sporozoites were incubated at 0.1, 1, 10 and 100 µg/mL of PTS and PTSO at 37 °C. A sample without compound was used as negative control and as positive control containing absolute ethanol to kill *Eimeria*. All treatments were performed in triplicate. The viability of sporozoites was determined at 0.5, 1, 2 and 4 h using propidium iodide (PI) (Acros Organics, Geel, Belgium) solution. To this aim, 90 µL of each sample solution containing sporozoites was mixed with 10 µL of PI solution previously prepared (500 µg PI/mL PBS), gently mixed and incubated for 10 min at 20 °C. Finally, 20 µL of each concentration was placed in a hemocytometer and sporozoites emitting red fluorescence were counted in a fluorescence/inverted microscope (Motic AE31E, Hong Kong, China). Laser excitation was 545 nm and emission 605 nm. Sporozoites emitting red fluorescence in the same manner as sporozoites from positive control were considered dead, while those without red fluorescence were considered viable.

In addition, PTS and PTSO were tested in MDBK cells to evaluate their possible cytotoxic effect. Cells were seeded in sterile 96-well plates at high density (1.4 × 10^4^ cells/well) and incubated at 37 °C with 5% CO_2_ for 24 h to allow cell adhesion. Increasing concentrations of PTS and PTSO (0.1, 0.5, 1, 5, 10, 50 and 100 µg/mL) were added in the corresponding wells and incubated for 72 h. The effect of each compound on MDBK was evaluated using a colorimetric technique with Sulforhodamine-B (SRB) [29]. Optical density values were determined at 490 nm, using a microplate reader (Multiskan EX, Thermo Electron Corporation, Minnesota, USA). The assessment of absorbance was obtained using the “SkanIt” RE 5.0 for Windows v.2.6 (Thermo Labsystems, Waltham, MA, USA).

### 2.5. Cell Cultures Infection

#### 2.5.1. Experiment 1: Inhibition of Sporozoites Invasion Capability

MDBK cells were grown overnight in 24-well plates at an initial density of 5 × 10^4^ cells per well in completed medium to allow their adhesion to the base of the well. Additionally, 5 × 10^4^ sporozoites were incubated at 0.1, 1 and 10 µg/mL of PTS or PTSO solutions for 1 h. A dilution of sporozoites without any treatment using the same medium containing DMSO and DMEM was used as positive control. All assays were performed in triplicate. After incubation, sporozoites were washed two times in PBS. The resultant pellet of each replicate was dissolved in DMEM and added to MDBK cells, and non-infected cells were used as negative control. After infection, cells were incubated for 24 h at 41 °C. Next, cells were carefully washed twice with PBS and detached with 200 µL trypsin that was later eliminated from these cells by centrifugation (1500× *g* for 5 min), and the pellets were resuspended in 500 μL DMSO and stored at −80 °C until DNA extraction.

In parallel, the same experiment was conducted during 72 h after infection, and cell viability was determined by SRB method as previously described.

#### 2.5.2. Experiment 2: Preventive Effect of Allium Compounds against Sporozoite Invasion

Before the infection with sporozoites, MDBK cells were seeded at a density of 5 × 10^4^ cells in 24-well plates and grown overnight at 37 °C and 5% CO_2_ to allow their adhesion to the base of the well. The next day, cells were covered by medium containing PTS or PTSO at different concentrations up to the highest values obtained in the cytotoxicity test that did not reduce more than 85–90% cell viability. In this way, the concentrations used for each compound were 0.1, 1, 5 and 10 µg/mL for PTS and 0.1, 1, 10 and 50 µg/mL for PTSO. All experiments included a positive control consisting of MDBK cells without previous treatment with PTS or PTSO. After 24 h, supernatants were removed and cells were washed twice with PBS. Then, a dilution of 5 × 10^4^ sporozoites was added to the cells, and they were incubated at 41 °C for 24 h, including a negative control with uninfected cells. All assays were performed in triplicate. Finally, cells were detached with trypsin, washed by centrifugation, and resuspended in DMSO and stored at −80 °C until DNA extraction. Again, the same experiment was conducted during 72 h after parasite addition and cell viability was determined by SRB method, as previously described.

### 2.6. Determination of Invasion Efficiency by qPCR

#### 2.6.1. DNA Extraction from Purified Oocysts and Samples

Purified *Eimeria* oocysts were used to obtain pure *Eimeria* DNA. Sporulated oocysts suspended in PBS were broken by adding an equal volume of autoclaved glass beads and vortexing for 3 min to disrupt the oocysts. Then, using the HigherPurity Tissue DNA Extraction kit (Canvax, Valladolid, Spain), DNA extraction from purified oocysts and MDBK cells from Experiments 1 and 2 were carried out following the instructions provided by the manufacturer. DNA extracted from oocysts and cells was stored at −20 °C for further use.

#### 2.6.2. Standard Curve

In order to generate a standard curve, purified *Eimeria* DNA was amplified by conventional PCR in a 2720 Thermal Cycler (Applied Biosystems, Singapore) using primers ACE-F (GCAGTCCGATGAAAGGTATTTG) and ACE-R (GAAGCGAAATGTTAGGCCATCT) targeting the sequence Ac-AD18-953 of *E. acervulina*, according to SCARdb database [30], to generate gene fragment with a product size of 103 bp [31]. The reaction volume (25 µL) contained 2 µL of DNA template, 2 µL (10 µM) of each primer, 12.5 µL of AmpliTaq Gold 360 Master Mix (Thermo Fischer Scientific, Vilnius, Lithuania) and 6.5 µL of Ultrapure nuclease-free water (Thermo Fischer Scientific, Bremen, Germany). Cycling condition consisted of 5 min at 95 °C, followed by 35 cycles of 10 s at 95 °C, 10 s at 62 °C and 10 s at 72 °C and a final extension step for 10 min at 72 °C. PCR product was run on 1.5% agarose gel to verify the exclusive presence of the expected fragment, and the corresponding band was excised and purified using Gen Elute™ PCR Clean-Up Kit (Sigma-Aldrich, Madrid, Spain). Purified PCR product was cloned in a TA cloning vector using TA Cloning^®^ Kits (Invitrogen, Darmstadt, Germany). The recombinant vector was cloned into *E. coli* and cultured overnight at 37 °C. Recombinant plasmids from the positive *E. coli* clones were extracted using Pure Link™ HQ Mini Plasmid Purification Kit (Invitrogen, Darmstadt, Germany) and were quantified using Qubit 4 Fluorometer (Invitrogen, Darmstadt, Germany). Copy numbers of recombinant plasmids were calculated from the concentration and size of the plasmids [32]. Finally, ten-fold serial dilutions of recombinant plasmids were prepared in nuclease-free water to obtain a range containing 10^7^ to 10^1^ plasmids/µL that were used for preparing the standard curves for absolute quantification in each experiment.

#### 2.6.3. Determination of Invasion Efficiency by qPCR

With the aim of detecting intracellular *Eimeria* DNA in MDBK cells that were harvested with sporozoites, qPCR was conducted on a Bio-Rad CFX Connect Real-Time PCR Detection System (Bio-Rad, Feldkirchen, Germany). For each sample, qPCR reactions consisted of 10 μL SYBR Green^®^ master mix (Thermo Scientific, Dreieich, Germany), 1 μL of forward and reverse primers 10 µM, 5 μL of DNA template and 3 μL nuclease-free water to obtain a final volume reaction of 20 μL. Each reaction was amplified in triplicate. A non-template control (NTC) consisting of nuclease-free water was added to each assay. Reaction conditions were 1min at 95 °C, followed by 35 cycles of 10 s at 95 °C, 10 s at 62 °C and 10 s at 72 °C. A melting curve was performed from 60 °C to 95 °C with heating rate of 0.1 °C per second, followed by one extension cycle of 30s at 50 °C.

The copy number of each sample was calculated based on the slope and intercept generated by the corresponding standard curve using qPCR software CFC manager v.3.1 (Bio-Rad Laboratories).

In vitro percentage of inhibition of sporozoite invasion for each *Allium* compound (%I_SIA_) was calculated with the following formula [33]:

%I_SIA_ = 100 × (1−(n° of gene copies of treated samples/nº of gene copies of untreated samples))

### 2.7. Statistical Analyses

Statistical analysis and figures were generated with GraphPad prism 8.0 software (GraphPad Software Inc., San Diego, CA, USA). Shapiro–Wilk normality tests were used to determine normal distribution of data. A two-way ANOVA test was used for comparison of viability of sporozoites considering concentrations of the compounds and time points. A one-way ANOVA test supplemented with Tukey’s post hoc was used for evaluation of statistically significant difference between number of parasite genomes, percentage of inhibition of sporozoite invasion and viability of cells. Differences were considered statistically significant when *p* > 0.05.

## 3. Results

### 3.1. Cytotoxicity of PTS and PTSO Compounds in Sporozoites and MDBK Cells

**Figure 1:**
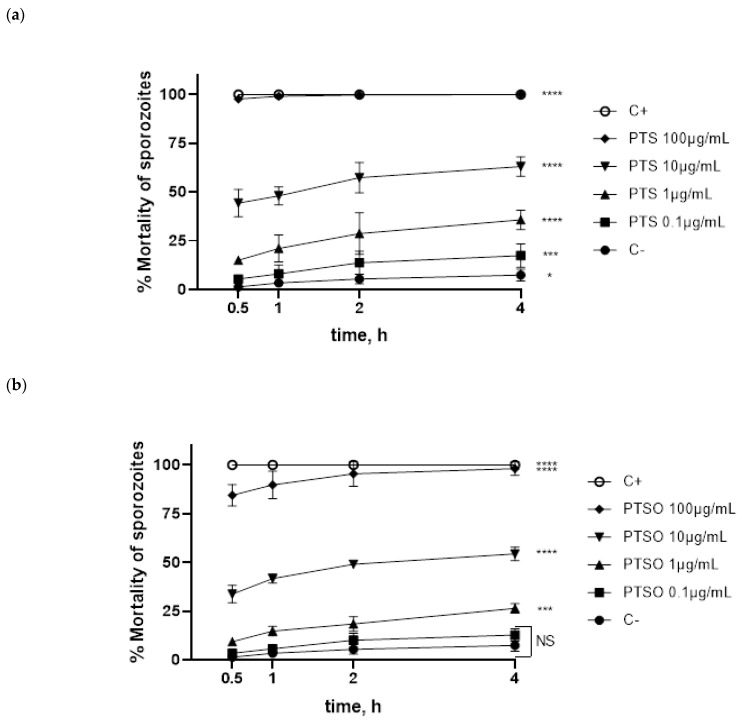
Mortality percentage of *E. acervulina* sporozoites over time (0.5, 1, 2 and 4 h) incubated at different concentrations of PTS (**a**) or PTSO (**b**). * *p* < 0.05; *** *p* < 0.001; **** *p* < 0.0001; NS, non-significant.

Sporozoite mortality increased within time in a dose-dependent way when incubated in each compound. Parasites showed higher sensibility to PTS, increasing mortality when compared to negative control from 0.1 µg/mL, while PTSO reduced the viability of parasites from 1 µg/mL onwards (Figure 1). After 4 h exposure, LD_50_ values of PTS and PTSO in sporozoites viability were 6.6 µg/mL and 8.6 µg/mL, and mortality percentages were 63.0% and 44.7%, respectively.

Regarding the toxicity results of each compound in MDBK cells, IC_50_ value for PTS was 26.3 µg/mL, while none of the PTSO concentrations tested reached the 50% of death population in this cell line. The concentration values of compounds that did not affect more than 10-15% to cell viability were 10 µg/mL for PTS and 50 µg/mL for PTSO, so these were the highest concentrations used for each compound in the performed assays.

### 3.2. In Vitro Anticoccidial Sensitivity Assays for qPCR

The standard curve showed a correlation (r^2^) value of 0.998 and a PCR efficiency of 99.8% for Experiment 1 and a (r^2^) of 0.989 and PCR efficiency of 102.6% for Experiment 2. Melting curve analysis showed a single melting peak with Tm value of 81.6 °C in both experiments.

### 3.3. Inhibition of Sporozoite Invasion Capability

**Figure 2:**
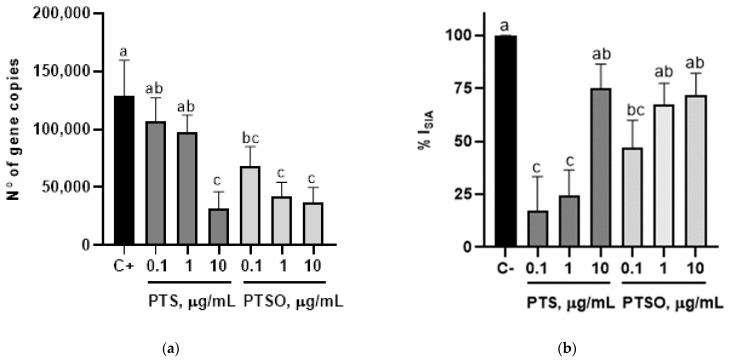
Effect of compound concentration on the estimated abundance of sporozoites, as estimated from qPCR: (**a**) number of *E. acervulina* gene copies detected in MDBK cells 24 h after the infection with sporozoites incubated at different PTS or PTSO concentrations; (**b**) percentage of inhibition of sporozoite invasion in MDBK cells when *E. acervulina* sporozoites were incubated at different PTS or PTSO concentrations; C+, MDBK cells infected with *E. acervulina* sporozoites that were not incubated with the compounds. Different letters between columns indicate significant differences at *p* < 0.05.

The incubation of sporozoites at 0.1, 1 and 10 µg/mL of PTSO reduced the copy number of the parasites in MDBK cells in all cases (6.8 × 10^4^ ± 9.61 × 10^3^; 4.2 × 10^4^ ± 7.3 × 10^3^ and 3.7 × 10^4^ ± 7.7 × 10^3^, respectively) compared to the positive control (1.29 × 10^5^ ± 3.07 × 10^4^). On the other hand, the incubation of sporozoites with PTS significantly reduced the cell invasion at a concentration of 10 µg/mL (3.2 × 10^4^ ± 8.30 × 10^3^) but not at 0.1 and 1 µg/mL. The highest percentages of inhibition of sporozoite invasion were obtained at 10 µg/mL of PTS (75.2% ± 6.44) and 1 and 10 µg/mL of PTSO (67.6% ± 5.65; 71.7% ± 6.03) (Figure 2).

**Figure 3:**
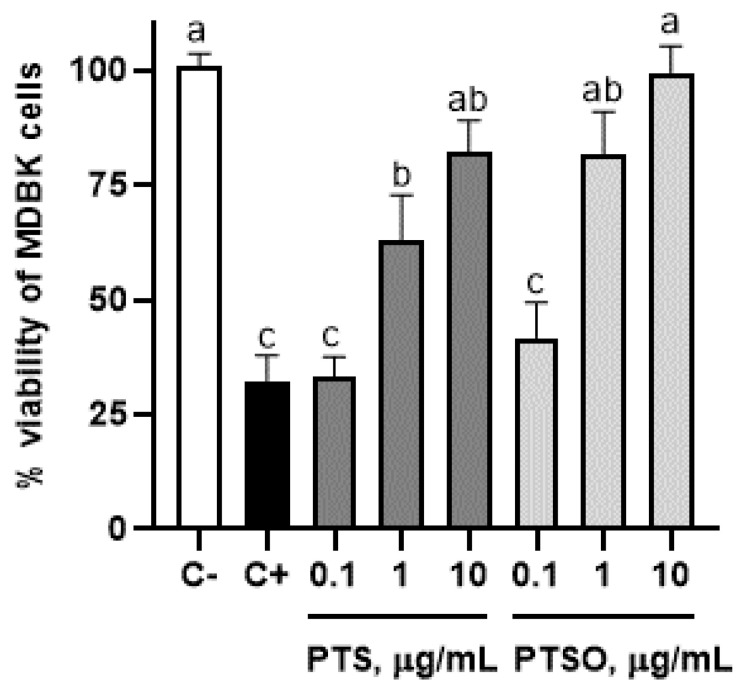
Viability percentage of MDBK cells infected with *E. acervulina* sporozoites that were previously incubated at different concentrations of PTS or PTSO. Different letters between columns indicate significant differences at *p* < 0.05.

The viability of MDBK cells after 72 h of infection with *E. acervulina* sporozoites incubated at different concentrations of PTS and PTSO are shown in Figure 3. Those MDBK cells that were infected with sporozoites incubated at 10 µg/mL of PTS and 1 and 10 µg/mL of PTSO showed similar viability percentages as unchallenged cells. However, viability of MDBK cells infected with 1 µg/mL of PTS obtained higher viability percentage values than those obtained in positive control (Figure 3).

### 3.4. Preventive Effect

**Figure 4:**
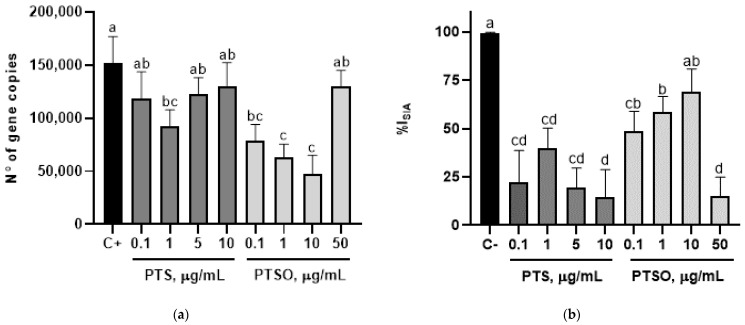
Effect of pre-incubation with PTS and PTSO on MDBK cells, estimated by qPCR: (**a**) number of *E. acervulina* gene copies detected in MDBK cells incubated at different PTS or PTSO concentrations 24 h before the infection with sporozoites; (**b**) percentage of inhibition of sporozoite invasion in MDBK cells incubated at different concentrations of PTS or PTSO before the infection with *E. acervulina* sporozoites. Different letters between columns indicate significant differences at *p* < 0.05.

The incubation of MDBK cells with PTS and PTSO reduced the number of gene copies of sporozoites significantly, showing the best results at the concentration 1 µg/mL of PTS (9.2 × 10^4^ ± 9.28 × 10^3^) and 0.1, 1 and 10 µg/mL of PTSO (7.8 × 10^4^ ± 9.03 × 10^3^; 6.3 × 10^4^ ± 7.10 × 10^3^ and 4.7 × 10^4^ ± 1.03 × 10^4^) compared to positive control (1.5 × 10^5^ ± 1.4 × 10^4^). Nevertheless, only PTSO at a concentration of 10 µg/mL was able to increase the percentage of inhibition of the cell invasion up to 69.5% ± 6.78, reaching similar values to those obtained in non-infected cells (Figure 4).

The viability of MDBK cells infected with sporozoites compared with those uninfected cells incubated at similar concentrations of PTS was lower at all concentrations tested. However, PTSO was able to prevent cellular death of infected MDBK cells at a concentration of 1 and 10 µg/mL, obtaining similar viability percentages to those uninfected cells incubated at the same PTSO concentrations (Table 1).

## 4. Discussion

The anticoccidial potential of the phytogenic compounds is based on their capability to reduce oocyst excretion through reaction with cytoplasmic membranes causing coccidian cell death, the impairment of the different stages of the parasite life cycle including the inhibition of the invasion, replication or development of parasites inside the enterocytes of chickens and repairing epithelial injuries through the upregulation of epithelial turnover [34,35]. In addition to current drugs, several studies have reported the effects of plant extracts, essential oils and phytogenic compounds in some of these aspects of coccidiosis control [36]. In this study, the inhibitory effects of PTS and PTSO on the viability of sporozoites and their capability for cell invasion were analyzed.

The exposure of sporozoites to different PTS or PTSO concentrations reduced their viability in a dose-dependent way. These results are similar to those obtained in a previous study in which sporozoites of *E. acervulina* were treated with a mix of 1:2 of both compounds, and viability was reduced up to 71% after 4 h of exposure [37]. Nevertheless, their results showed higher toxicity of PTS/PTSO than the ones obtained in our study, probably due to a synergistic effect when testing both OSCs together. However, PTS and PTSO were more effective in reducing sporozoite viability than allicin, which decreased sporozoite viability from 50 µM (equivalent to 8.1 mg/mL) onwards [38]. Compared to antibiotics, PTS and PTSO showed higher effectiveness against sporozoites than the ionophorus compounds salinomycin and lasalocid [39].

In our first experiment, *E. acervulina* sporozoites were previously incubated at different concentrations of PTS and PTSO before challenging MDBK cells. The reduction in the efficiency of the invasion observed in the sporozoites treated with PTS could be due in part to the death of the parasites due to the high toxicity that, according to our results, this biomolecule presents on the parasites. However, after one hour of exposure to PTS, parasite mortality was less than the reduction in invasion efficiency, indicating some additional inhibitory effect of PTS on the sporozoite. In the same way, despite the effect of PTSO on sporozoite viability at a concentration of 1 µg being softer than PTS, it was high enough to prevent sporozoites penetration in MDBK cells, suggesting, in the same way as PTS, changes in sporozoites membrane that directly affected their penetration capability. This hypothesis could be supported by the fact that similar compounds such as allicin and their derivates have shown the inhibitory effect against parasites enzymes necessary to play an essential role in the their pathogenicity., The yield of thiol groups to the cysteine residue of the active site prevent adhesion and penetration into the cells [40]. Although the effect of PTS and PTSO on these proteins and other apical complex structures have never been described, a similar enzymatic process could be the reason for the inhibitory effect of PTSO. Nevertheless, despite the functional properties of allicin that have been widely described, it presents low stability that could influence the results obtained in different studies depending on the assay conditions [41,42]. Our results are comparable to those obtained in a similar study in which *E. tenella* sporozoites were incubated at different concentrations of allicin, obtaining reductions of MDBK cell invasion from 54% (for 1.8 ng/mL) to 99% (for 180 mg/mL). In contrast, an in vitro study in which *E. tenella* sporozoites were incubated at different concentrations of oregano or garlic extract reported a 93% inhibition of parasite invasion when incubated with oregano essential oil at a concentration of 100 μg/mL, while garlic essential oil reached a maximum inhibition of 70% after 24 h at 50 μg/mL [43]. As mentioned above, the term garlic extract encompasses different types of compounds at different concentrations that directly influence the results. In addition, despite the fact that their study was carried out with *Alliaceae* extracts, they do not contain the PTS and PTSO compounds derived from onions.

On the other hand, PTSO showed a strong capability to exert a preventive effect on cell invasion at the lowest concentrations, but higher concentrations of each compound did not prevent parasite penetration into the cells. Considering the dose ranges at which each compound did not affect cell viability, it could be suggested that at low concentrations, PTSO exerted an immunomodulatory effect that inhibits the expression of genes related to inflammation and oxidative damage, while the highest concentrations cause metabolism changes to protect cell survival, reducing the preventive ability of external damage. Some studies have shown that MDBK cells are able to produce cytokines depending on the culture conditions and when stimulated by exposure to viruses [44,45], suggesting that an immune response could also be involved in the anticoccidial effect of these metabolites. Moreover, in previous studies, PTSO has shown an immunomodulatory in vitro effect in Caco-2 and THP-1 cells by reducing the production of pro-inflammatory mediators and downregulating mitogen-activated protein kinases (MAPKs) signaling pathways involved in intestinal epithelial barrier integrity [46]. In addition, RNA-sequencing demonstrates that PTSO-PTS induces the expression of a number of genes involved in antioxidant responses in intestinal epithelial cells (IEC) during exposure to antigens from the parasite *Trichuris muris* and strongly suppresses pathways related to immune and inflammatory signaling [47]. Furthermore, plant extracts with antioxidant activity, such as *Tulbaghia violacea*, *Vitis vinifera* and *Artemisia afra,* have been demonstrated to reduce the negative consequences of coccidiosis in in vivo studies [48]. In addition, the inclusion of a mix of polyphenols and an extract of aromatic plants in the diet of coccidia-challenged chickens reduced lesion scores in the *duodenum*, *jejunum* and *ceca* after 25 days, probably due to the antioxidant capacity of the compounds [49]. Despite the fact that we did not measure the expression of those genes related to oxidative damage in this study, we also suggest a similar mechanism of action that could explain the inhibition profile at different doses obtained in our experiment.

Finally, other in vitro studies have reported the efficacy of phytogenic extracts to inhibit sporozoite cell invasion. Carvacrol, curcumin and *Echinacea purpurea* extract have been shown to reduce in vitro parasite cell invasion when cells were incubated at different concentrations of the compounds [50]. In the same way, another study reported an inhibitory effect of the invasion when MDBK cells were incubated with saponins, carvacrol and thymol [51]. Nevertheless, in both studies, phytogenic compounds were not removed before the addition of parasites, thus making it difficult to discern whether the compounds could have a preventive or direct effect. However, new studies are necessary to understand the protective effects of these OSCs from onion and their influence on MDBK cells’ gene expression and the reduction of oxidative stress. Moreover, the preventive effect of these compounds should be further confirmed in in vivo studies.

## 5. Conclusions

PTS and PTSO derivatives from onion showed strong antiparasitic activity against *E. acervulina* parasites, reducing their ability to penetrate cells. Moreover, in MDBK cells, PTS, particularly PTSO, exerted a preventive effect against sporozoite invasion, although additional in vitro studies are needed to verify their mechanism of action and in vivo assays to confirm these results in chickens.

## Figures and Tables

**Table 1:** Comparison between viability of non-infected or infected MDBK cells that were incubated during 24 h at the same concentrations of PTS or PTSO.

		PTS, µg/mL				PTSO, µg/mL			
	Control	0.1	1	5	10	0.1	1	10	50
**MDBK ^1^**	100.90	95.65	103.50	100.30	85.40	99.88	99.36	97.38	88.60
**MDBK + E ^2^**	32.55	34.43	47.09	40.18	23.85	73.38	93.48	95.96	40.91
**SEM**	2.28	2.438	2.181	2.28	2.28	2.181	2.28	2.438	2.28
***p*-value**	<0.0001	<0.0001	<0.0001	<0.0001	<0.0001	<0.0001	0.113	0.9994	<0.0001

^1^ MDBK, non-infected cells; ^2^ MDBK + E, infected cells with *E. acervulina* sporozoites.

## Data Availability

The data presented in this study are available on request from the corresponding author.

## References

[1] Geetha M., Palanivel K.M. (2018). A review on poultry coccidiosis. Int. J. Curr. Microbiol. Appl. Sci..

[2] Chapman H.D. (2014). Milestones in avian coccidiosis research: A review. Poult. Sci..

[3] Noack S., Chapman H.D., Selzer P.M. (2019). Anticoccidial drugs of the livestock industry. Parasitol. Res..

[4] Adhikari P., Kiess A., Adhikari R., Jha R. (2020). An approach to alternative strategies to control avian coccidiosis and necrotic enteritis. J. Appl. Poult. Res..

[5] Quiroz-Castañeda R.E., Dantán-González E. (2015). Control of avian coccidiosis: Future and present natural alternatives. BioMed Res. Int..

[6] Peek H.W., Landman W.J.M. (2011). Coccidiosis in poultry: Anticoccidial products, vaccines and other prevention strategies. Veter Q..

[7] Suresh G., Das R.K., Brar S.K., Rouissi T., Ramirez A.A., Chorfi Y., Godbout S. (2018). Alternatives to antibiotics in poultry feed: Molecular perspectives. Crit. Rev. Microbiol..

[8] Wink M. (2018). Plant secondary metabolites modulate insect behavior-steps toward addiction?. Front. Physiol..

[9] Yang C., Chowdhury M.A.K., Hou Y., Gong J. (2015). Phytogenic compounds as alternatives to in-feed antibiotics: Potentials and challenges in application. Pathogens.

[10] Vidanarachchi J.K., Mikkelsen L.L., Sims I., Iji P.A., Choct M. (2005). Phytobiotics: Alternatives to antibiotic growth promoters in monogastric animal feeds. Recent Adv. Anim. Nutr. Aust..

[11] Sharifi-Rad M., Mnayer D., Tabanelli G., Stojanović-Radić Z.Z., Sharifi-Rad M., Yousaf Z., Vallone L., Setzer W.N., Iriti M. (2016). Plants of the genus Allium as antibacterial agents: From tradition to pharmacy. Cell. Mol. Biol..

[12] Kothari D., Lee W.-D., Niu K.-M., Kim S.-K. (2019). The genus allium as poultry feed additive: A review. Animals.

[13] Khalil A.M., Yasuda M., Farid A.S., Desouky M.I., Mohi-Eldin M.M., Haridy M., Horii Y. (2015). Immunomodulatory and antiparasitic effects of garlic extract on Eimeria vermiformis-infected mice. Parasitol. Res..

[14] Foroutan-Rad M., Tappeh K.H., Khademvatan S. (2017). Antileishmanial and immunomodulatory activity of *Allium sativum*(Garlic): A review. J. Evid. Based Integr. Med..

[15] Fujisawa H., Suma K., Origuchi K., Kumagai H., Seki T., Ariga T. (2008). Biological and chemical stability of garlic-derived allicin. J. Agric. Food Chem..

[16] Putnik P., Gabrić D., Roohinejad S., Barba F.J., Granato D., Mallikarjunan K., Lorenzo J.M., Bursać Kovačević D. (2019). An overview of organosulfur compounds from *Allium* spp.: From processing and preservation to evaluation of their bioavailability, antimicrobial, and anti-inflammatory properties. Food Chem..

[17] Cascajosa-Lira A., Ortega A.I.P., Guzmán-Guillén R., Cătunescu G.M., de la Torre J.M., Guillamón E., Jos Á., Fernández A.M.C. (2021). Simultaneous determination of Allium compounds (Propyl propane thiosulfonate and thiosulfinate) in animal feed using UPLC-MS/MS. Food Chem. Toxicol..

[18] Guillamón E., Andreo-Martínez P., Mut-Salud N., Fonollá J., Baños A. (2021). Beneficial effects of organosulfur compounds from *Allium cepa* on gut health: A systematic review. Foods.

[19] Peinado M.J., Ruiz R., Echávarri A., Aranda-Olmedo I., Rubio L.A. (2013). Garlic derivative PTS-O modulates intestinal microbiota composition and improves digestibility in growing broiler chickens. Anim. Feed Sci. Technol..

[20] Rabelo-Ruiz M., Ariza-Romero J.J., Zurita-González M.J., Martín-Platero A.M., Baños A., Maqueda M., Valdivia E., Martínez-Bueno M., Peralta-Sánchez J. (2021). *Allium*-based phytobiotic enhances egg production in laying hens through microbial composition changes in ileum and cecum. Animals.

[21] Abad P., Arroyo-Manzanares N., Ariza J.J., Baños A., García-Campaña A.M. (2021). Effect of allium extract supplementation on egg quality, productivity, and intestinal microbiota of laying hens. Animals.

[22] Sánchez C.J., Martínez-Miró S., Ariza J.J., Madrid J., Orengo J., Aguinaga M.A., Baños A., Hernández F. (2020). Effect of *Alliaceae* extract supplementation on performance and intestinal microbiota of growing-finishing pig. Animals.

[23] Rabelo-Ruiz M., Teso-Pérez C., Peralta-Sánchez J.M., Ariza J.J., Martín-Platero A.M., Casabuena-Rincón Ó., Vázquez-Chas P., Guillamón E., Aguinaga-Casañas M.A., Maqueda M. (2021). *Allium* extract implements weaned piglet’s productive parameters by modulating distal gut microbiota. Antibiotics.

[24] Sorlozano-Puerto A., Albertuz-Crespo M., Lopez-Machado I., Gil-Martinez L., Ariza-Romero J.J., Maroto-Tello A., Baños-Arjona A., Gutierrez-Fernandez J. (2021). Antibacterial and antifungal activity of propyl-propane-thiosulfinate and propyl-propane-thiosulfonate, two organosulfur compounds from *Allium cepa*: In vitro antimicrobial effect via the gas phase. Pharmaceuticals.

[25] Sorlozano-Puerto A., Albertuz-Crespo M., Lopez-Machado I., Ariza-Romero J.J., Baños-Arjona A., Exposito-Ruiz M., Gutierrez-Fernandez J. (2018). In vitro antibacterial activity of propyl-propane-thiosulfinate and propyl-propane-thiosulfonate derived from *Allium* spp. against gram-negative and gram-positive multidrug-resistant bacteria isolated from human samples. BioMed Res. Int..

[26] Qi N., Liao S., Abuzeid A.M., Li J., Wu C., Lv M., Lin X., Hu J., Xiao W., Sun M. (2020). Effect of different floatation solutions on E. tenella oocyst purification and optimization of centrifugation conditions for improved recovery of oocysts and sporocysts. Exp. Parasitol..

[27] Castañón C.A.B., Fraga J.S., Fernandez S., Gruber A., Costa L.D.F. (2007). Biological shape characterization for automatic image recognition and diagnosis of protozoan parasites of the genus Eimeria. Pattern Recognit..

[28] Pastor-Fernández I., Pegg E., Macdonald S.E., Tomley F.M., Blake D.P., Marugán-Hernández V. (2019). Laboratory growth and genetic manipulation of *Eimeria tenella*. Curr. Protoc. Microbiol..

[29] Vichai V., Kirtikara K. (2006). Sulforhodamine B colorimetric assay for cytotoxicity screening. Nat. Protoc..

[30] Fernández S., Katsuyama Â.M., Kashiwabara A.Y., Madeira A.M.B.N., Durham A.M., Gruber A. (2004). Characterization of SCAR markers of Eimeria spp. of domestic fowl and construction of a public relational database (The Eimeria SCARdb). FEMS Microbiol. Lett..

[31] Kundu K., Kumar S., Banerjee P.S., Garg R. (2020). Quantification of Eimeria necatrix, E. acervulina and E. maxima genomes in commercial chicken farms by quantitative real time PCR. J. Parasit. Dis..

[32] Dhanasekaran S., Doherty T.M., Kenneth J. (2010). Comparison of different standards for real-time PCR-based absolute quantification. J. Immunol. Methods.

[33] Thabet A., Zhang R., Alnassan A.-A., Daugschies A., Bangoura B. (2017). Anticoccidial efficacy testing: In vitro Eimeria tenella assays as replacement for animal experiments. Veter Parasitol..

[34] Burrell A., Tomley F.M., Vaughan S., Marugan-Hernandez V. (2020). Life cycle stages, specific organelles and invasion mechanisms of *Eimeria* species. Parasitology.

[35] López-Osorio S., Chaparro-Gutiérrez J.J., Gómez-Osorio L.M. (2020). Overview of poultry eimeria life cycle and host-parasite interactions. Front. Veter Sci..

[36] El-Shall N.A., El-Hack M.E.A., Albaqami N.M., Khafaga A.F., Taha A.E., Swelum A.A., El-Saadony M.T., Salem H.M., El-Tahan A.M., AbuQamar S.F. (2021). Phytochemical control of poultry coccidiosis: A review. Poult. Sci..

[37] Kim D.K., Lillehoj H.S., Lee S.H., Lillehoj E.P., Bravo D. (2012). Improved resistance to *Eimeria acervulina* infection in chickens due to dietary supplementation with garlic metabolites. Br. J. Nutr..

[38] Coppi A., Cabinian M., Mirelman D., Sinnis P. (2006). Antimalarial activity of allicin, a biologically active compound from garlic cloves. Antimicrob. Agents Chemother..

[39] Mehlhorn H., Pooch H., Raether W. (1983). The action of polyether ionophorous antibiotics (monensin, salinomycin, lasalocid) on developmental stages of Eimeria tenella (Coccidia, Sporozoa) in vivo and in vitro: Study by light and electron microscopy. Z. Für Parasitenkd.

[40] Waag T., Gelhaus C., Rath J., Stich A., Leippe M., Schirmeister T. (2010). Allicin and derivates are cysteine protease inhibitors with antiparasitic activity. Bioorganic Med. Chem. Lett..

[41] Shang A., Cao S.-Y., Xu X.-Y., Gan R.-Y., Tang G.-Y., Corke H., Mavumengwana V., Li H.-B. (2019). Bioactive compounds and biological functions of garlic (*Allium sativum* L.). Foods.

[42] Borlinghaus J., Albrecht F., Gruhlke M.C.H., Nwachukwu I.D., Slusarenko A.J. (2014). Allicin: Chemistry and biological properties. Molecules.

[43] Sidiropoulou E., Skoufos I., Marugan-Hernandez V., Giannenas I., Bonos E., Aguiar-Martins K., Lazari D., Blake D.P., Tzora A. (2020). In vitro anticoccidial study of oregano and garlic essential oils and effects on growth performance, fecal oocyst output, and intestinal microbiota in vivo. Front. Veter Sci..

[44] Elsasser T.H., Caperna T.J., Ward P.J., Sartin J.L., Steele B.P., Li C., Kahl S. (2007). Modeling growth factor activity during proinflammatory stress: Methodological considerations in assessing cytokine modulation of IGF binding proteins released by cultured bovine kidney epithelial cells. Domest. Anim. Endocrinol..

[45] Fredericksen F., Carrasco G., Villalba M., Olavarría V.H. (2015). Cytopathic BVDV-1 strain induces immune marker production in bovine cells through the NF-κB signaling pathway. Mol. Immunol..

[46] Vezza T., Algieri F., Garrido-Mesa J., Utrilla M.P., Rodríguez-Cabezas M.E., Baños A., Guillamón E., García F., Rodríguez-Nogales A., Galvez J. (2019). The immunomodulatory properties of propyl-propane thiosulfonate contribute to its intestinal anti-inflammatory effect in experimental colitis. Mol. Nutr. Food Res..

[47] Zhu L., Myhill L.J., Andersen-Civil A.I., Thamsborg S.M., Blanchard A., Williams A.R. (2022). Garlic-derived organosulfur compounds regulate metabolic and immune pathways in macrophages and attenuate intestinal inflammation in mice. Mol. Nutr. Food Res..

[48] Naidoo V., McGaw L.J., Bisschop S.P.R., Duncan N., Eloff J.N. (2008). The value of plant extracts with antioxidant activity in attenuating coccidiosis in broiler chickens. Veter Parasitol..

[49] Alhotan R.A., Abudabos A. (2019). Anticoccidial and antioxidant effects of plants derived polyphenol in broilers exposed to induced coccidiosis. Environ. Sci. Pollut. Res..

[50] Burt S.A., Tersteeg-Zijderveld M.H.G., Jongerius-Gortemaker B.G.M., Vervelde L., Vernooij J.C.M. (2013). In vitro inhibition of Eimeria tenella invasion of epithelial cells by phytochemicals. Veter Parasitol..

[51] Felici M., Tugnoli B., Ghiselli F., Massi P., Tosi G., Fiorentini L., Piva A., Grilli E. (2020). In vitro anticoccidial activity of thymol, carvacrol, and saponins. Poult. Sci..

